# The Role of the Mannich Reaction in Nitrogen Migration during the Co-Hydrothermal Carbonization of Bovine Serum Albumin and Lignin with Various Forms of Acid–Alcohol Assistance

**DOI:** 10.3390/molecules28114408

**Published:** 2023-05-29

**Authors:** Qiang Zhang, Kai Mu, Bo Zhao, Linlin Yi

**Affiliations:** 1 School of Resource and Environmental Engineering, Wuhan University of Science and Technology, Wuhan 430081, China; 2 State Key Laboratory of Coal Combustion, School of Energy and Power Engineering, Huazhong University of Science and Technology, Wuhan 430074, China

**Keywords:** co-hydrothermal carbonization, Mannich reaction, acid–alcohol assistance, nitrogen migration, model compounds, biomass hydrochar, carbon-neutral fuel

## Abstract

Co-hydrothermal carbonization (co-HTC) of N-rich and lignocellulosic biomass is a potential way to produce hydrochar with high yield and quality, but the nitrogen will also enrich in a solid product. In this study, a novel co-HTC with acid–alcohol assistance is proposed, and the model compounds bovine serum albumin (BSA) and lignin were used to investigate the role of the acid–alcohol-enhanced Mannich reaction in nitrogen migration. The results showed that the acid–alcohol mixture could inhibit nitrogen enrichment in solids and the order of the denitrification rate was acetic acid > oxalic acid > citric acid. Acetic acid promoted solid-N hydrolysis to NH_4_^+^ while oxalic acid preferred to convert it to oil-N. More tertiary amines and phenols were generated with oxalic acid–ethanol addition and then formed quaternary-N and N-containing aromatic compounds through the Mannich reaction. In the citric acid–ethanol–water solution, NH_4_^+^ and amino acids were captured to form diazoxide derivatives in oil and pyrroles in solids through both nucleophilic substitution and the Mannich reaction. The results are able to guide biomass hydrochar production with the targeted regulation of nitrogen content and species.

## 1. Introduction

In recent years, climate change caused by the greenhouse effect has triggered a string of ecological, environmental, and social issues. As a representative greenhouse gas, CO_2_ emissions from fossil fuels reached 34.8 billion tons in 2020 [1]. Carbon emission reduction has become a common goal in the international community to promote sustainable development. Biomass is a renewable resource possessing the ability to reabsorb the carbon emitted during combustion, the thermal utilization of which is regarded as carbon neutral or carbon negative [2]. Needless to say, the application of biofuels instead of fossil fuels is a promising way to realize carbon emission reduction.

Different from lignocellulosic biomass consisting of cellulose, hemicelluloses, and lignin, N-rich biomass such as sewage sludge and microalgae is mainly composed of proteins and lipids [3,4]. The direct thermal utilization of N-rich biomass is not recommended on account of high moisture and the presence of nitrogenous pollutants [5]. Hydrothermal carbonization (HTC) is a cheap and simple method for wet organic waste’s conversion to carbon-enriched hydrochar with favorable energy density [6,7]. In the meantime, many proteins enter the liquid phase, reducing the nitrogen yield in hydrochar to 30% or lower [8]. Due to the lower carbohydrate content, which is an ideal component for hydrochar production, N-rich biomass keeps its solid yield at a low level, and the average value such as in sludge is less than 60% [9]. Moreover, the combustion performance and mechanical properties of hydrochar obtained from single N-rich biomass hydrolysis are mediocre [10]. Thus, co-HTC of N-rich and lignocellulosic biomass is proposed to improve the yield and quality of hydrochar.

He et al. [11] achieved organic enrichment in sludge hydrochar by adding various forms of agricultural biomass. Lignin was considered the major contributor to enhancing the heating value and combustion performance of the hydrochar. Liu et al. [12] adjusted the organics composition of a rape straw–microalgae mixture with acid treatment. Higher cellulose content was conducive to hydrochar production and spherical structure formation. Zhang et al. [13] found that sludge co-HTC with pine wood had better performance in hydrochar yield and carbon retention, and the synergistic coefficients were 8.41% and 13.09%, respectively. However, solid-N also increased from 21.85% to 60.82%, at most when more pine wood participated. The same phenomenon was observed for the co-HTC of a waste lettuce–protein mixture, a swine manure–sawdust mixture, and a glucose–spirulina platensis mixture, in which the maximum nitrogen content of hydrochar reached 80%, raising the risk of N-pollutants release in subsequent thermal utilization [14,15,16]. More attention should be paid to nitrogen conversion during co-HTC, owing to nitrogen re-solidification. The Maillard reaction between carbohydrate derivatives and amino acids as well as the Mannich reaction between phenols, N-containing compounds, and aldehydes are the critical interactions associated with nitrogen migration in the co-hydrothermal process [16,17]. In the Maillard reaction, monosaccharides derived from hemicellulose and cellulose captured amino acids to form intermediate imines, which directly participated in hydrochar formation or were converted into N-heterocyclic compounds through dehydration and ring closure [18,19]. The phenols from lignin degradation preferred to react with amines/ammonia and aldehydes to form N-containing groups in solid via the Mannich reaction [17]. Due to the high degradation temperature of lignin (above 523 K) [20], the Mannich reaction was weak in the regular temperature range of HTC (453~523 K) and received less attention during co-HTC.

In recent studies, different liquid catalysts such as organic acids, protic solvents, and oxidants have been applied for hydrochar upgradation. The catalysts not only increased the severity of the HTC reaction and facilitated the depolymerization of biomass but also provided additional substrates for repolymerization to form hydrochar [21,22]. The interactions must be strengthened in catalytic systems with complex nitrogen conversion. Our previous studies proposed the technology of acid–alcohol-assisted co-HTC and achieved hydrochar denitrification at equivalent or more output. With acid–alcohol assistance, the Mannich reaction became remarkable even at 453 K and the effects on nitrogen migration could not be ignored. Furthermore, it was difficult to distinguish the effects of the enhanced Mannich reaction in real biomass because the Maillard reaction frequently coexisted.

In this study, co-HTC of bovine serum albumin (BSA) and lignin in various acid–alcohol–water solutions was conducted to investigate the effect of the enhanced Mannich reaction on nitrogen migration as well as the role of different acid–alcohol solvents. The results will guide the production of biomass hydrochar with the targeted regulation of nitrogen content and species.

## 2. Results and Discussion

### 2.1. Nitrogen Distribution in Acid–Alcohol-Assisted Co-HTC Products

**Figure 1:**
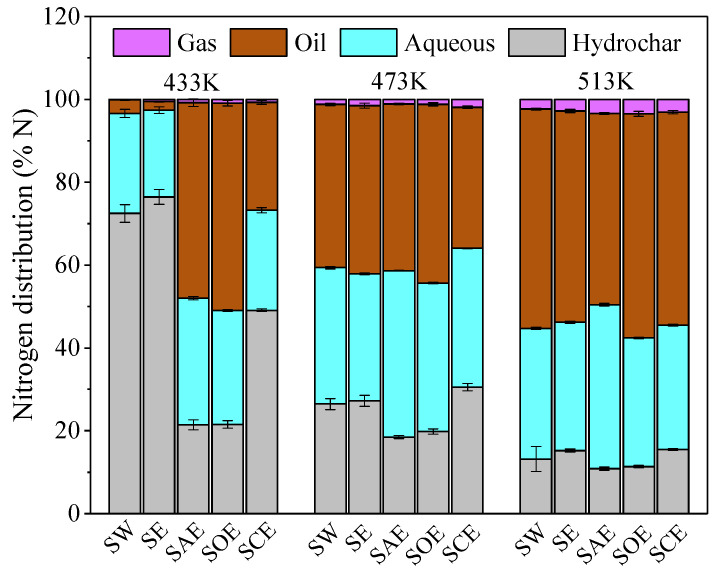
Nitrogen distribution in different products during the co-HTC of BSA and lignin.

The nitrogen distribution in different products during the co-HTC of BSA and lignin with various solvents is displayed in Figure 1. In the temperature range of 433 K to 513 K, most of the nitrogen migrated into the aqueous and oil products, and the nitrogen contents in gas calculated by difference were less than 3.5%. A sharp decrease in nitrogen content from 72.4% to 13.2% in the SW (hydrolysis with water) hydrochar, accompanied by the same trend in BSA, showed the advantage of the HTC process in N-rich biomass denitrification. The aqueous-N was unstable at higher temperatures and converted to oil-N. The 0.8~4.1% growth of SE (hydrolysis with ethanol) hydrochar-N from aqueous-N and oil-N conversion was the result of ethanol addition. More phenols were generated from lignin decomposition and captured both amine and ammonia in solid via the Mannich reaction [23].

The nitrogen contents in the hydrochar dropped dramatically at 433 K after acid–alcohol solvent assistance, and the decrements were 51.1%, 50.9%, and 23.3% for SAE (hydrolysis with the acetic acid–ethanol mixture), SOE (hydrolysis with the oxalic acid–ethanol mixture), and SCE (hydrolysis with the citric acid–ethanol mixture), respectively. The peptide bond in the proteins can be rapidly catalytically broken in an acidic reaction medium [24], resulting in amine generation through decarboxylation and ammonia through deamination. As the hydrolysis efficiency of the BSA increased at higher temperatures, the effect of acids on hydrochar denitrification gradually became weak. At 513 K, the nitrogen contents in the SAE and SOE hydrochar were still less than that in SW even though the BSA had decomposed completely in the absence of acids. It was inferred that acetic acid and oxalic acid could prevent N-containing compounds enrichment in hydrochar. Acetic acid demonstrated the best performance in denitrification as the nitrogen content decreased by 2.3~51.1%. When citric acid was used instead of acetic acid, 4.6~27.6% of the nitrogen in the aqueous and oil phase was re-solidified in the hydrochar. Citric acid was considered to promote the Mannich reaction for the higher nitrogen content in the SCE hydrochar than in SE.

### 2.2. Properties of Hydrochar and N-Species in Hydrochar

**Figure 2:**
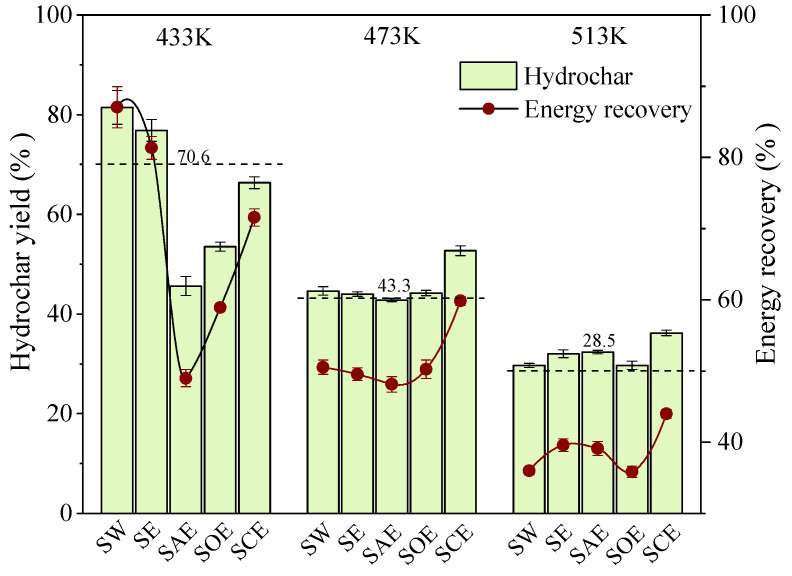
Effect of the acid–alcohol solvent on the hydrochar yield and energy recovery.

The yield and energy recovery of the hydrochar are illustrated in Figure 2, and the dotted lines represent the yield summation of BSA and lignin in separate hydrolysis. The yield of the SE hydrochar was 1.1%~10.8% higher than the dotted lines, confirming the existence of the Mannich reaction during co-HTC. Compared with SW, the yield and energy recovery of the SE hydrochar decreased by 4.6% and 5.7%, respectively, at 443 K, but the trend then gradually began to increase with the temperature rising. There was reason to believe that ethanol performed better at low temperatures in enhancing the Mannich reaction for the maximum growth of nitrogen content in the SE hydrochar at 433 K. Therefore, enhancing the Mannich reaction was not the same as increasing the yield and energy recovery. The organic species as well as the degradation degree at that time needed to be considered as ethanol was able to accelerate some organics solvolysis [25]. Due to the catalytic degradation of BSA, the addition of acetic acid led to the largest decline in the energy-related performance of the hydrochar at 433 K, and the decrements of yield and energy recovery were 35.8% and 38.1%, respectively, followed by oxalic acid (27.9% and 28.1%, respectively) and citric acid (15.1% and 15.5%, respectively). Similarly, the trend reversed with temperature, especially for the hydrochar assisted with the citric acid–ethanol mixture, which possessed the highest yield and energy recovery, 6.6% and 8.1% more than those of SW at 513 K, respectively. Combined with the nitrogen distribution in Figure 1, it was found, of great significance, that the acetic acid–ethanol and oxalic acid–ethanol mixtures could remove nitrogen from the hydrochar without mass and energy loss, while the citric acid–ethanol mixture preferred to improve the fuel properties of the hydrochar. He et al. [26] compared the characteristics of citric-acid- and acetic-acid-treated hydrochar from the co-HTC of food waste digestate and yard waste and found that the former had advantages in terms of combustion behavior and energy properties. As a tricarboxylic acid, citric acid may have a high capacity to accelerate the hydrolysis of organics into smaller fragments and then enhance the repolymerization process. Moreover, citric acid may undergo nucleophilic substitution with amino acids, because more than one carboxyl group in one citric acid molecule was found capable of reacting with protein by cross-linking.

**Figure 3:**
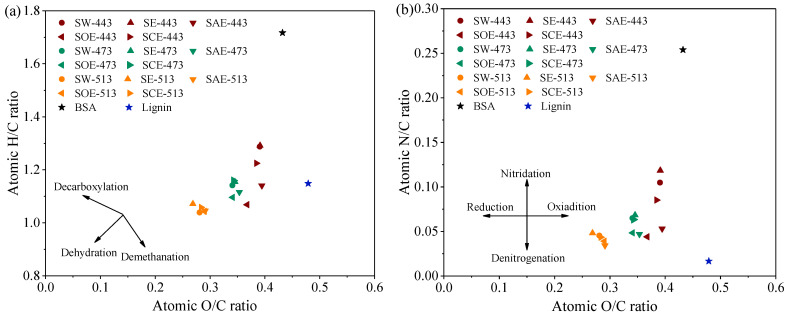
Effect of the acid–alcohol solvent on the hydrochar (**a**) H/C ratios and (**b**) N/C ratios.

As the hydrothermal temperature increased, a steady reduction in the H/C, N/C, and O/C atomic ratios was observed, as shown in Figure 3a,b. High temperatures were in favor of hydrochar quality upgradation through dehydration, decarboxylation, and denitrification. The addition of ethanol led to hydrochar nitridation, but the nitrogen would be swiftly removed in an acidic environment. All of the acid–alcohol mixtures showed the ability of hydrochar denitrification even though the nitrogen content increased in the SCE hydrochar. It meant that citric acid could boost carbon retention in the hydrochar with less nitrogen re-solidification than non-catalytic runs. At the same time, the H/C atomic ratio of SAE, SOE, and SCE hydrochar decreased obviously, symbolizing the intensification of dehydration. The Mannich reaction enhanced by the acid–alcohol solvent could improve hydrochar properties, especially at low temperatures. In general, HTC at high temperatures seemed unwise for N-rich biomass for the goal of solid fuel production, because of lower yield and energy recovery. The low temperature was also inappropriate due to the poor carbonized degree of the hydrochar. Acid–alcohol assistance could strengthen a series of reactions including organics depolymerization, fragments recombination with the Mannich reaction, and repolymerization, resulting in hydrochar generation at low temperatures with both high quality and yield.

**Figure 4:**
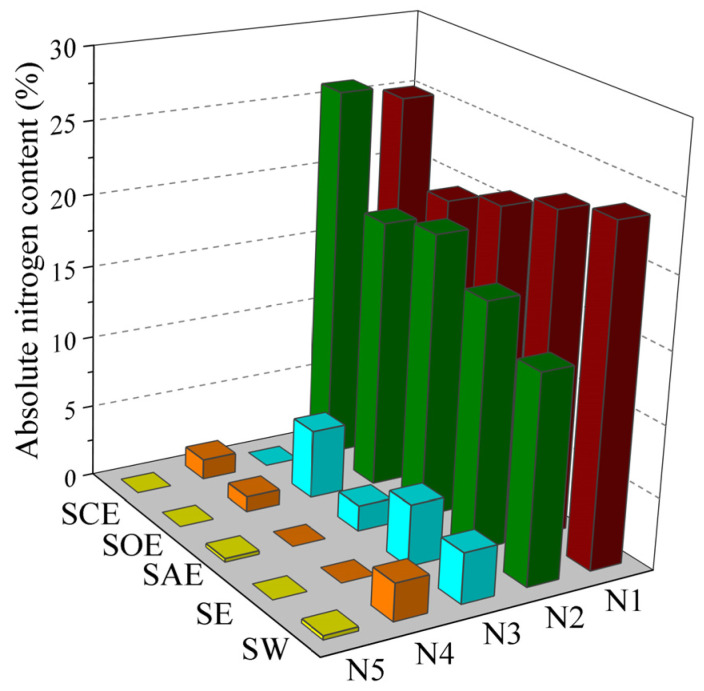
N-species in different hydrochar at 473 K (N1: protein-N, N2: pyrrole-N, N3: quaternary-N, N4: amine-N/pyridine-N, and N5: inorganic-N).

The N-species and absolute contents in the different hydrochar at 473 K are presented in Figure 4. It was found that protein-N accounted for the highest nitrogen content (23.6%) in the SW hydrochar, followed by pyrrole-N (14.5%), quaternary-N (3.5%), amine-N/pyridine-N (2.6%), and inorganic-N (0.3%). With ethanol addition, protein-N and amine-N/pyridine-N decomposed and migrated to liquid and then converted to pyrrole-N and quaternary-N via the Mannich reaction. More of the phenols were generated from the lignin in the ethanol solution, which could combine with pyridine to form quaternary-N by polymerization and the condensation cyclization reaction [27]. The absolute content of protein-N decreased to 20.9% and 19.6% in the SAE and SOE hydrochar, respectively, and the content of pyrrole-N increased to 19.7% and 18.7%, indicating that acetic acid and oxalic acid promoted protein-N transformation to pyrrole-N by depolymerization and cyclization. The addition of oxalic acid increased the quaternary-N content by 1.3%, but it was labile in the acetic acid solution and converted to pyrrole-N. The citric acid–ethanol mixture may have inhibited the decomposition of protein-N as the absolute content of protein-N was nearly the same in the SW and SCE hydrochar. During HTC, the dehydration of citric acid may lead to trans-aconitic acid generation, which further gave itaconic anhydride by decarboxylation. The isomerization of itaconic anhydride formed citraconic anhydride, preserving some amino acids via a reversible preservation reaction [28]. The highest content of pyrrole-N was 26.1% and appeared in the SCE hydrochar, showing that the citric acid–ethanol mixture re-solidified the aqueous-N and oil-N in solid in the form of pyrroles.

### 2.3. Nitrogen Distribution in Aqueous and Oil Products

**Figure 5:**
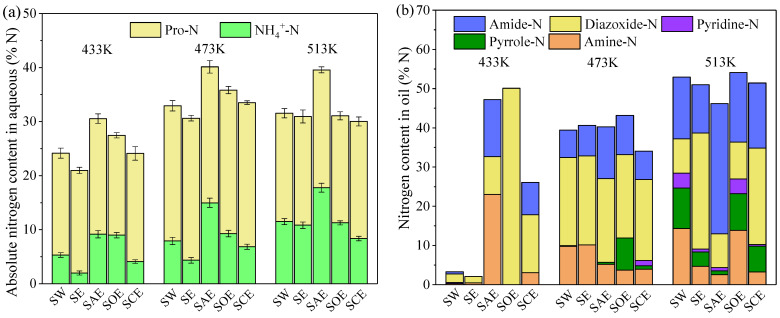
Nitrogen distribution in (**a**) aqueous products and (**b**) oil products.

According to one study [29], the N-species in liquid were classified as NH_4_^+^-N, NO_2_^−^-N, NO_3_^−^-N, and Pro-N in aqueous products and organic-N in oil products (as shown in Figure 5a,b). The total concentration of NO_2_^−^-N and NO_3_^−^-N was less than 0.1%, and the transformation was ignored in this study. The diazoxide-N here referred to a series of derivatives including piperazinones, pyrimidines, and so on. It was observed that acids and ethanol had little impact on solute Pro-N conversion compared with the solid protein. The decomposition of Pro-N may be a reversible reaction and is mainly affected by temperature. As the temperature went up from 433 K to 513 K, the content of NH_4_^+^-N for SW increased steadily from 5.3% to 11.5%, with it being produced by the deamination of protein [30]. In the meantime, abundant amine-N, amide-N, and diazoxide-N arose in oil, and then partial diazoxide-N decomposed to other N-containing compounds with temperature. The diazoxide-N which had two N atoms in a six-membered ring may result from the dimerization of amino acids [31]. Compared with SW, the content of NH_4_^+^-N in SE liquid products decreased by 0.6~3.4%, along with the increment of amide-N in 473 K and diazoxide-N in 513 K. In the protonic environment provided by ethanol, NH_4_^+^ could react with the carboxyl existing in the amino acid to form amino acid amide compounds through the Mannich reaction, which were then converted to diazoxide-N by intramolecular dehydration and cyclization. Although the amine-N contents were close at 473 K in the SW and SE oil products, the variation trend became different. About 80% of amine-N in the SE oil products was detected in the form of a tertiary amine; however, the contents of primary and secondary amines approached 50% for SW. The decline in amine-N in the SE oil products indicated that the enhanced Mannich reaction also conduced to tertiary amine formation and its conversion to hydrochar-N as quaternary-N.

Similar to hydrochar-N, the distribution of liquid-N still relied on the types of organic acids. The highest contents of NH_4_^+^-N at different temperatures were 9.2~17.7% and appeared in SAE aqueous products. Acetic acid promoted the deamination of amines as the amine-N content gradually decreased with temperature. The diazoxide-N derived was unstable in a high temperature acidic environment, leading to an amide-N content increase of 20.8%. The amide-N was detected mainly in the form of ethyl acetimidate and acetamide, and it was supposed that acetic acid was inclined to crack pyrimidine derivatives, which possessed two N atoms in the 1 and 3 sites. In the SOE liquid products, the content of NH_4_^+^-N was 5.7~6.8% less than SAE despite more solid protein being decomposed, as shown in Figure 4, demonstrating that the oxalic acid–ethanol mixture made solid-N and NH_4_^+^-N migrate to oil-N. Different from acetic acid, oxalic acid was likely to promote the dehydration and cyclization of amino acids, because of large amounts of diazoxide-N, which were identified as cyclic dipeptides by GC-MS generated at 433 K. With the rise in temperature, the diazoxide-N was unstable and constantly transformed into amine-N, pyrrole-N, and pyridine-N rather than amide-N. The renascent amine-N was mainly a tertiary amine and then formed quaternary-N by the Mannich reaction. Abundant urea and intermediate ammonium carbamate were detected in the SOE amide-N, which may be the reason for NH_4_^+^-N capture. Urea was also found in the SCE amide-N; however, the NH_4_^+^-N was more inclined to form diazoxide-N in oil or pyrrole-N in hydrochar. In view of the best performance to solidify nitrogen in hydrochar, citric acid may capture nitrogen in other ways. It was reported that during HTC, citric acid could produce citraconic anhydride or citrate anions; the former may react with NH_4_^+^ and the latter may undergo nucleophilic substitution with amino acids to form intermediates [28,32]. In citric acid–ethanol–water solution, the N-containing intermediates were selectively converted to diazoxide-N as the content still kept a high level at 513 K.

### 2.4. Other Organic Components in Oil

To identify the reaction mechanism of nitrogen migration, the ring structures in the oil products were simply classified, and the relative contents were computed, as shown in Table 1. High temperatures were conducive to the ring structures opening and the branched or oxygenous structures’ removal, as the corresponding relative contents decreased sharply. With ethanol addition, at 473 K and 513 K when abundant nitrogen was released to liquid, the relative content of chain structures decreased by 13.57~20.45%, along with the increment of cyclobenzene and branched and oxygenous structures. Combined with Figure 5b, it was indicated that the enhanced Mannich reaction facilitated amine-N and amide-N conversion to ring structures, particularly at 513 K to form two rings which had both diazoxide and benzene ring structures. Among the three types of acids, acetic acid possessed the strongest degrading ability on ring structures while oxalic acid and citric acid contributed to cyclization. The ratio of branched/oxygenous structures was less in the SCE oil than in the SOE, indicating that more O atoms were directly connected to the carbon in the ring. Considering that almost all of the ring structures in the SCE oil contained oxygenous groups and the relative content of the chain structures was only 27.42% even at 513 K, it was speculated that citric acid could participate in the cyclization reaction as a reactant more than just a solvent.

**Figure 6:**
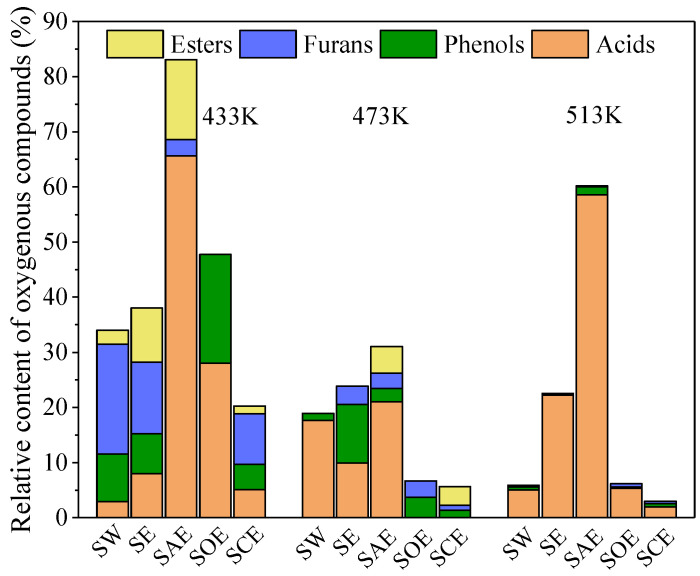
Relative contents of different oxygenous structures in oil.

The relative contents of other oxygenous compounds in the oil (except for N-O heterocyclic compounds) were further refined, as shown in Figure 6. Acids, phenols, furans, and esters were the four main oxygenous components during the co-HTC of BSA and lignin. Aldehydes and ketones have also been reported as important intermediates during HTC [12], but none were detected in this study. Perhaps the aldehydes and ketones mainly came from the degradation of hemicellulose and cellulose, a small amount of which from lignin might combine with N-containing compounds to form amides and diazoxide derivatives. The relative content of acids in the SAE oil was far more than others, and both acetic acid and other organic acids were detected. Acetic acid may promote acid production by deaminizing amino acids or cracking the C-O/C=O groups in the ring. Compared with oxalic acid and citric acid, the cyclization ability of acetic acid was quite weak and only formed esters, which would decompose at higher temperatures and release acids. The relative content of furans decreased with acids and ethanol addition, which were the key precursors to form N-heterocyclic compounds [18]. The relative contents of phenols were higher in the SE and SOE oil at 433 K, and then decreased with temperature. The Mannich reaction between the phenols and N-containing compounds may be the reason for the high content of benzene ring structures and branched structures in the SE and SOE oil. The total relative contents of oxygenous structures were kept lowest in the SCE oil, indicating that citric acid could strengthen the reactions between carbon- and nitrogen-containing groups such as aminomethylation, dehydration condensation, and so on.

### 2.5. Possible Path of Nitrogen Migration in Various Acid–Alcohol-Assisted Co-HTC Experiments

**Figure 7:**
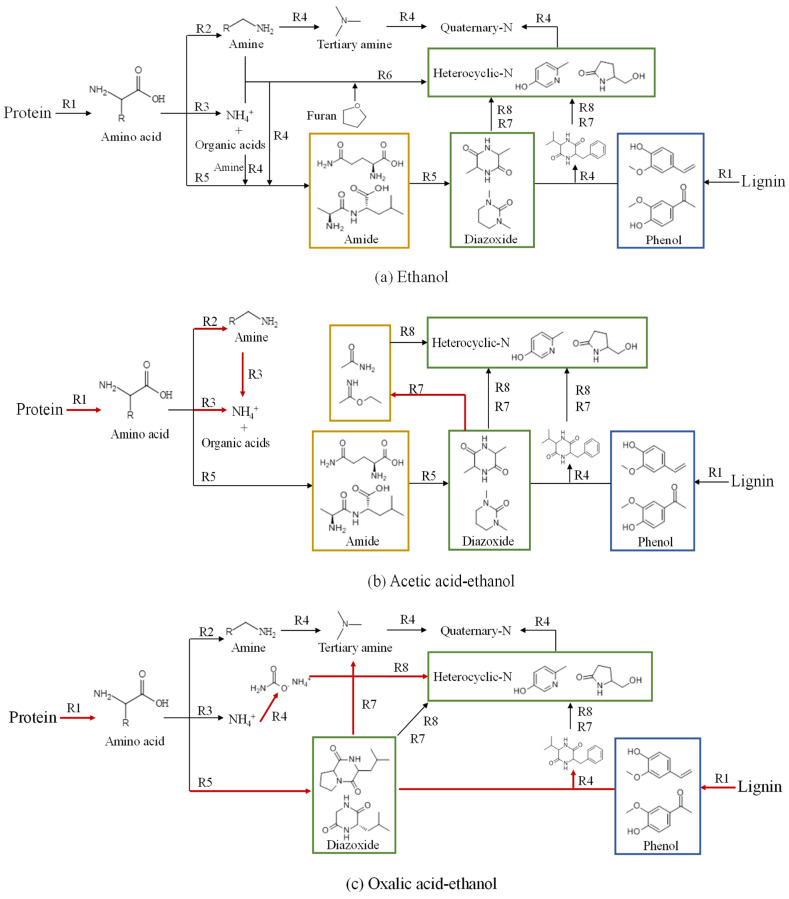

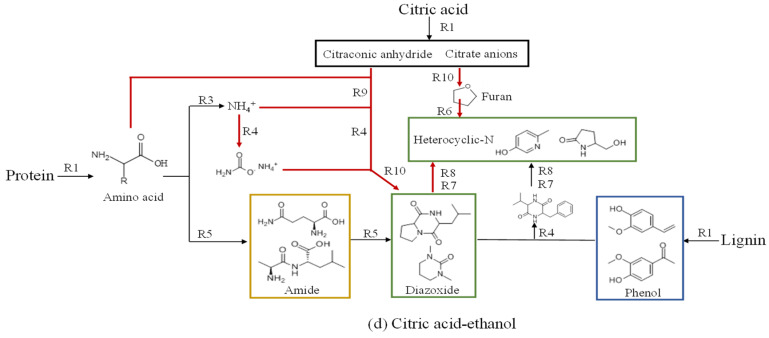
Possible path of nitrogen migration during co-HTC with acid–alcohol assistance: (**a**) ethanol, (**b**) acetic acid–ethanol, (**c**) oxalic acid–ethanol, and (**d**) citric acid–ethanol.

Based on the results of the nitrogen distribution in the solid and liquid products, the possible path of nitrogen migration in various acid–alcohol-assisted co-HTC experiments was established, as shown in Figure 7. In the ethanol–water solution, protein and lignin were hydrolyzed (R1) to produce amino acids and phenols, respectively. The soluble amino acids may undergo three pathways of transformation: (1) decarboxylation (R2), which produced amines which were then converted to tertiary amines and further quaternary-N by the Mannich reaction (R4); (2) deamination, which produced NH_4_^+^ and organic acids; (3) dehydration condensation (R5), which produced amides. The amides may also generate from the Mannich reaction (R4) between organic acids and amines or between amines/NH_4_^+^ and amino acids and continued dehydration condensation (R5) to form diazoxide derivatives. The diazoxide derivatives reacted with phenols to form aromatic compounds via the Mannich reaction (R4). However, the diazoxide structures were unstable at high temperatures and transformed into stable heterocyclic-N (pyridine or pyrrole derivatives) through ring cleavage (R7), recombination, and polymerization (R8). The nitridation (R6) of furans with amines and NH4+ may be another way to produce heterocyclic-N, part of which would be converted to quaternary-N through the Mannich reaction (R4). Both heterocyclic-N and quaternary-N participated in solid formation and generated corresponding N-containing compounds retained in the hydrochar.

In the different acid–alcohol–water solution systems, acetic acid promoted the hydrolysis (R1) of protein and the deamination (R3) of amino acids and amines to release NH_4_^+^. The Mannich reaction (R4) to capture the amines and NH_4_^+^ was inhibited and more solid-N migrated to aqueous-N. Ring cleavage (R7) was also enhanced with acetic acid and diazoxide derivatives (especially pyrimidine derivatives) decomposed to ethyl acetimidate and acetamide, part of which formed heterocyclic-N in the hydrochar through recombination and polymerization (R8). Oxalic acid also promoted the hydrolysis (R1) of protein but preferred to produce diazoxide derivatives (especially two-ring structures) through deep dehydration condensation of amino acids (R5), meaning that oxalic acid could remove solid-N to oil-N. At high temperatures, ring cleavage (R7) of diazoxide derivatives produced tertiary amines, providing resources for quaternary-N generation. A portion of NH_4_^+^ was converted to ammonium carbamates, the intermediates of urea production, and then formed heterocyclic-N in oil through recombination and polymerization (R8). Moreover, more phenols were generated in the oxalic acid–ethanol–water solution, which was in favor of N-containing aromatic compounds’ preparation. Differently, in the citric acid–ethanol–water solution, hydrolysis (R1) of protein was inhibited or not promoted, and the NH_4_^+^ was captured by the Mannich reaction (R4). The citraconic anhydride and citrate anions derived from citric acid could also react with NH_4_^+^ (R4) and amino acids (nucleophilic substitution, R9) to obtain N-containing intermediates, which were likely to form diazoxide derivatives by dehydration and cyclization (R10). High content of diazoxide derivatives led to pyrroles formation in SCE hydrochar. Moreover, the nitridation (R6) of furans generated from citric acid dehydration and cyclization (R10) may be another reason for pyrroles production.

## 3. Materials and Methods

### 3.1. Materials

Bovine serum albumin and lignin were selected to represent the protein in N-rich biomass and the lignin component in lignocellulosic biomass, respectively. Before the co-HTC experiments, the hydrolysis efficiency of raw materials was measured at the designed temperatures (433 K, 473 K, and 513 K), and the results are shown in Table 2. The temperature had obvious significance in protein decomposition, whilst the hydrolysis efficiency of the lignin only increased by 12.8% from 433 K to 513 K. The ultimate analysis of raw materials is also listed in Table 2.

### 3.2. Co-HTC of BSA and Lignin

To distinguish the effects of acidity on the Mannich reaction, five types of solvent were employed, including pure water (namely SW), ethanol (namely SA), an acetic acid–ethanol mixture (namely SAE), an oxalic acid–ethanol mixture (namely SOE), and a citric acid–ethanol mixture (namely SCE). In each co-HTC experiment, 0.75 g BSA, 0.75 g lignin, 25 g water, and 0.15 g solvent were mixed in pressure tanks and heated at the designed temperature for 2 h. After the reactor cooled to room temperature, the solid and liquid mixtures were separated through extraction filtration. Before storage and testing, the hydrochar was dried at 378 K, and the liquid products were filtered by a 0.45 m PTFE filter membrane. Each experiment was repeated more than three times.

### 3.3. Analysis of Products from Co-HTC

An elemental analyzer (Elementar company, Germany) was used to analyze the C, H, N, and S contents in the hydrochar, and the O content was calculated by difference. The N-species in the hydrochar were decided by X-ray photoelectron spectroscopy (Axis-ultra dld-600 W, Japan). The nitrogen groups could be divided into five categories, including pyridine-N or amine-N (398.8 ev), protein-N (399.8 ev), pyrrole-N (400.2 ev), quaternary-N (401.4 ev), and inorganic-N (402.9 ev) [33,34]. Equations (1) and (2) were applied to calculate the energy recovery efficiency of the hydrochar.

HHV (MJ kg^−1^) = 0.3517C + 1.1626H + 0.1047S − 0.111O (1)

Energy recovery (%) = yield HHVhydrochar/(HHVfeedstock + HHVsolvent)(2)

The N-species in the liquid included inorganic nitrogen (NH_4_^+^-N, NO_2_^−^-N, and NO_3_^−^-N) in aqueous phase and organic nitrogen (TON), and the latter could be further classified as soluble proteins (Pro-N) in aqueous phase and N-containing compounds in oil phase (Oil-N). The concentrations were calculated using Equations (3) and (4). Total nitrogen (TN) was detected by a total organic carbon analyzer (multiN/C2100, Germany). The inorganic-N was determined by ion chromatography (ICS-6000, Metrohm 883), and the Pro-N was measured by the bicinchoninic acid method and a BCA Protein Assay Kit [35]. After concentration with a water bath nitrogen blowing instrument (ZGDCY-125), the N-species in the oil were detected by GC-MS (Agilent 7890A/5975 C, USA).

TON = TN − (NH_4_^+^-N + NO_2_^−^-N + NO_3_^−^-N) (3)

Oil-N = TON − Pro-N(4)

## 4. Conclusions

The acid–alcohol solvents had significant impacts on the Mannich reaction and nitrogen migration during co-HTC. At 473 K, acetic acid and oxalic acid removed about 8% of the nitrogen from the hydrochar without yield and energy loss. The Mannich reaction related to the phenols was enhanced in the ethanol and oxalic acid–ethanol solution, resulting in quaternary-N and aromatic compound generation. Citric acid increased the yield and energy recovery of the hydrochar by 6.1% and 9.3% at most, respectively, and re-solidified the NH_4_^+^ and oil-N in the hydrochar as pyrroles. This meant that the properties, nitrogen content, and species of the hydrochar could be controlled by acid–alcohol solvents to determine its utilization.

## Figures and Tables

**Table 1:** Relative contents of ring structures classification in oil products.

Sample	One Ring	Two Rings	Three Rings	Cyclobenzene	Branched	Oxygen
SW-433	18.79	53.91	13.35	28.38	52.22	83.54
SE-433	39.84	23.37	16.79	6.72	60.80	65.82
SAE-433	8.01	4.65	2.02	4.03	11.75	14.68
SOE-433	28.03	30.61	3.41	34.42	59.23	54.21
SCE-433	17.31	44.06	1.54	20.10	58.65	62.91
SW-473	18.50	28.04	-	9.36	33.95	43.36
SE-473	31.89	28.22	-	21.30	43.89	56.93
SAE-473	31.89	22.53	-	13.16	42.79	54.42
SOE-473	41.49	29.87	-	16.48	58.89	68.93
SCE-473	22.04	47.10	-	13.58	51.19	67.98
SW-513	23.58	19.53	0.20	8.37	26.93	40.52
SE-513	27.34	36.42	-	22.59	50.46	50.49
SAE-513	7.42	4.15	0.14	2.54	10.33	11.45
SOE-513	32.04	15.68	-	8.80	39.65	42.18
SCE-513	52.51	17.27	2.80	5.95	45.01	72.08

**Table 2:** Ultimate analysis and hydrolysis efficiency of the BSA and lignin.

Sample	Hydrolysis Efficiency (%)				Ultimate Analysis (wt%)			
	433 K	473 K	513 K	C	H	N	S	O ^a^
BSA	28.6	75.5	100	48.98	7.01	14.50	1.28	28.23
Lignin	30.1	37.8	42.9	56.91	5.44	1.10	0.20	36.35

^a^ calculated by difference.

## Data Availability

Not applicable.

## Sample Availability

Samples of the compounds are available from the authors.
